# Gut microbiota-targeted bioactive peptides as emerging axis in nutritional therapeutics: trends, challenges, and prospects

**DOI:** 10.3389/fnut.2026.1905173

**Published:** 2026-08-17

**Authors:** Brij Pal Singh, Prabhashis Bose, Jae-Young Je

**Affiliations:** 1 University Centre for Research and Development (UCRD), Chandigarh University, Mohali, Punjab, India; 2 Department of Food Science and Technology, University of Nebraska-Lincoln, Lincoln, NE, United States; 3 Nebraska Food for Health Center, University of Nebraska-Lincoln, Lincoln, NE, United States; 4 Major of Human Bioconvergence, Division of Smart Healthcare, Pukyong National University, Busan, Republic of Korea

**Keywords:** anti-inflammatory, bioactive peptides, dysbiosis, gut microbiota, gut-brain axis, immunomodulatory

## Abstract

The gut microbiota is a dynamic, complex microbial ecosystem that is fundamental to human health and influences metabolism, immunity, and susceptibility to disease. There is growing evidence that bioactive peptides (BAPs) are important regulators of gut microbiota composition and function. BAPs are short protein fragments liberated through enzymatic hydrolysis or microbial fermentation *in vitro* and during the digestion of food proteins *in vivo*. The biological activities of these BAPs, beyond their antioxidant, antihypertensive, and antimicrobial effects, include maintaining gut balance and supporting overall well-being. The relationship between BAPs and gut microbiota is two-way: peptides selectively promote beneficial microbes, and the microbiota enzymatically convert peptides into bioactive metabolites such as short-chain fatty acids and altered bile acids, or further hydrolyze them into shorter BAPs. This interaction between gut microbiota and BAPs has been shown to confer significant therapeutic benefits from combined nutritional interventions in the management of chronic diseases, including obesity, diabetes, inflammatory bowel diseases, and neurological diseases. This review examines the current state of gut microbiota-targeted BAPs, including their sources, properties, and mechanisms. It underscores their contribution to microbial diversity and metabolic activity, which support immune balance and the functioning of the gut-brain axis. In addition, it addresses novel ways to increase the activity of BAPs with respect to stability and delivery. Challenges such as peptide bioavailability, microbiome diversity, and a lack of clinical evidence are also critically discussed. Future perspectives focus on leveraging omics technologies and personalized nutrition to unlock the potential of BAPs for therapeutic and functional food applications. The review indicates the promising potential of BAPs as microbiota-based, innovative, and useful agents in nutritional therapy.

## 1 Introduction

The human gastrointestinal tract plays a key role in overall health and disease regulation, hosting a diverse and complex community of microorganisms, together known as the gut microbiota (1). This hidden ecosystem is essential for maintaining equilibrium in the body’s metabolic and immunological systems and for regulating the gut-brain axis (2, 3). In recent years, with advances in sequencing technologies and systems biology, the gut microbiota has ceased to be viewed as a passive resident and is now regarded as an active organ capable of influencing host physiology and adapting to numerous environmental conditions (4).

Food and its bioactive constituents play an important role in host health by shaping the gut microbiota, selectively promoting beneficial microbes, and modulating microbial composition and metabolic activity (5). Among them, bioactive peptides (BAPs), short chains of amino acids derived from food proteins, have emerged as promising bioactive compounds with immense potential in health and nutrition. Unlike conventional nutrients, BAPs exert specific physiological effects, including antimicrobial, anti-inflammatory, immunomodulatory, antidiabetic, anticancer, and antioxidant activities, among others, thereby influencing multiple parameters through their defined bioactivities (6–9).

In this regard, exploration of the relationship between BAPs and the gut microbiome offers new possibilities for the management of chronic diseases, the development of functional foods, and the advancement of personalized nutrition. Emerging evidence suggests a bidirectional relationship between BAPs and the gut microbiota. Peptides selectively influence microbial composition and metabolism, while microbial proteases transform dietary peptides into metabolites or smaller bioactive fragments with distinct activities. Microbiota-BAPs communication on the metabolic level offers the basis of innovative therapies against obesity, diabetes, inflammatory bowel diseases (IBDs), or even neurological diseases (10–12). However, the molecular mechanisms behind these interactions remain incompletely understood. Much of the evidence comes from preclinical models rather than well-controlled human studies.

Beyond their immediate biological influences, gut microbiota-specific BAPs have been of interest for their capacity to restore eubiosis and overcome dysbiosis. Eubiosis is a balanced, healthy gut microbiota that supports normal physiological functions, whereas dysbiosis is a disrupted microbial balance that may contribute to disease development (1, 13, 14). Well-characterized BAPs and BAP-rich foods can have selective effects, as they can increase the growth of beneficial microbial communities and inhibit the growth of harmful ones.

This review provides an updated, comprehensive overview of gut microbiota-targeted BAPs, integrating recent advances in peptide discovery, microbiota modulation, and host physiological regulation. The existing body of literature has described the biological functions of BAPs and their general interactions with the gut microbiota, but important knowledge gaps remain regarding the molecular mechanisms underlying peptide–microbiota crosstalk, their microbiota-mediated therapeutic effects across chronic diseases, and the technological strategies to improve peptide stability, delivery, and clinical translation. Limited attention has been given to the emerging roles of multi-omics technologies, precision nutrition, and personalized microbiome-based interventions in advancing peptide therapeutics. Therefore, this review critically summarizes current evidence on the reciprocal interactions between BAPs and the gut microbiota. It discusses their implications in gastrointestinal, metabolic, inflammatory, and neurological disorders, evaluates recent advances in delivery technologies and translational challenges, and identifies future research priorities for developing microbiota-targeted functional foods and nutritional therapies.

## 2 Gut microbiota function and dysbiosis

The human gastrointestinal tract harbors approximately 38 trillion microorganisms that collectively constitute the gut microbiota, one of the most metabolically active ecosystems in the human body (15). A healthy gut contains over one thousand distinct bacterial species, most of which are located in the large intestine’s lumen. In healthy adults, it is primarily composed of four main bacterial phyla: Firmicutes, Bacteroidetes, Actinobacteria, and Proteobacteria, with the first two together representing over 95% of the total microbial population. Beyond bacteria, the gut microbiota also includes viruses, fungi, archaea, and microeukaryotes (16, 17). The human gut microbiota is a dynamic, highly personalized ecosystem of microorganisms that plays a crucial role in digestion, metabolism, immune system regulation, and overall health and disease (18, 19).

**Figure 1:**
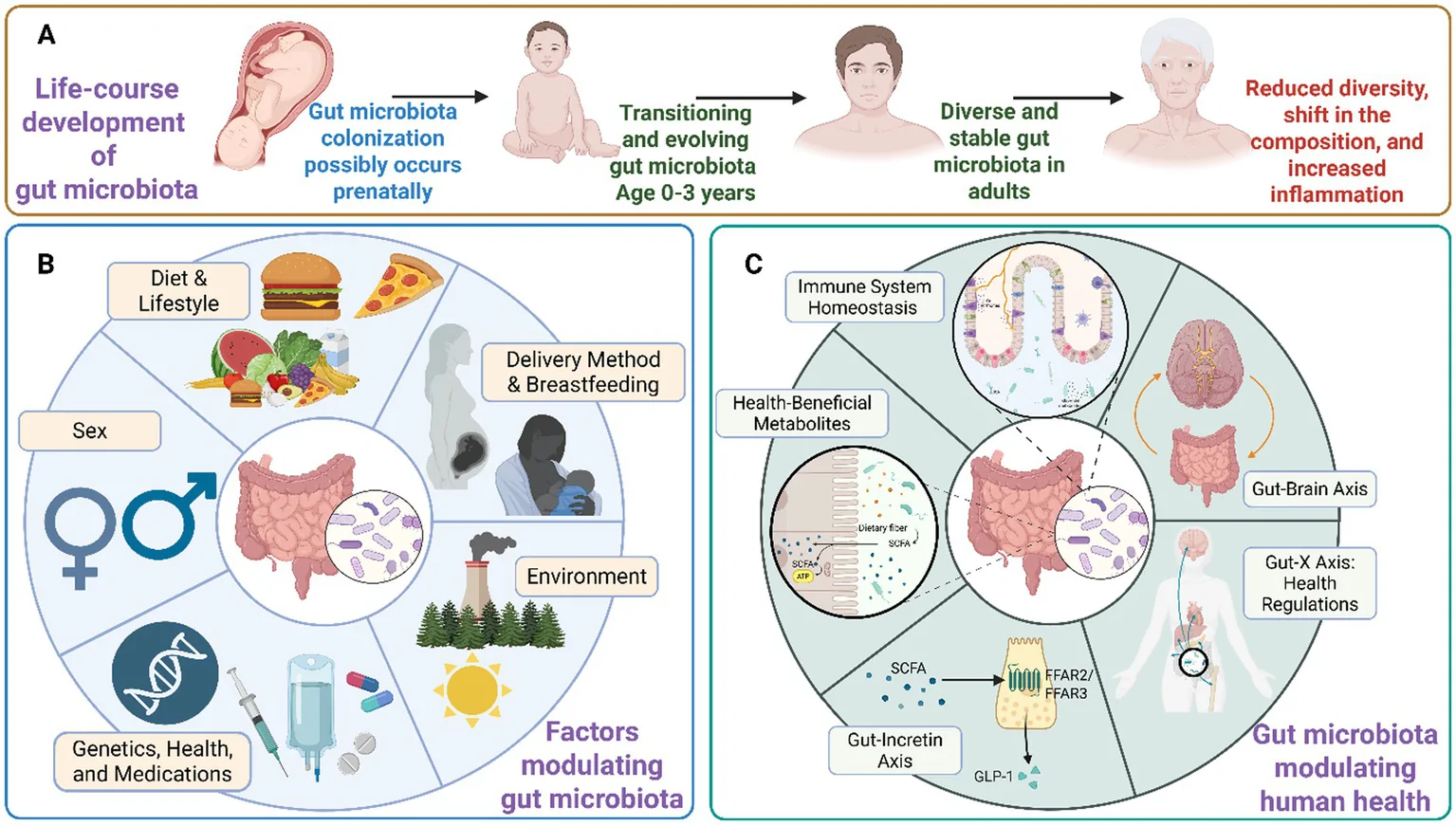
Life-course development of the gut microbiota, its modulating factors, and its contributions to human health. **(A)** Schematic representation of the temporal development of the human gut microbiota across the life course. **(B)** Overview of the principal host and environment associated factors that modulate gut microbiota composition and function, including diet and lifestyle, mode of delivery and breastfeeding, environmental exposures, host genetics, health status and medications, and biological sex. **(C)** Overview of the mechanisms through which the gut microbiota modulates host physiology and health.

Microbial colonization begins early in life, possibly even in the womb, and becomes established primarily by the ages of three to four years. After this developmental stage, the gut microbiota gradually diversifies and stabilizes throughout adulthood, until aging reduces diversity and resilience (20–22). While healthy individuals share a common “core” microbiota, the specific makeup of microbial species varies widely. Both modifiable and non-modifiable factors influence this variation. Modifiable factors include diet, antibiotic use, breastfeeding, and environment, while non-modifiable factors include genetics, birth method, geographic location, and age (21, 23, 24). The composition of the gut microbiota reflects a finely tuned balance shaped by an individual’s life course. It includes dominant bacterial phyla, key butyrate-producing genera, diet-responsive microbes, and taxa influenced by host genetics. These factors play a vital role in regulating human physiology and disease susceptibility. Therefore, the gut microbiota has recently been classified as a “vital organ” because of its complex and communicative connections with other organs through neural, endocrine, humoral, immunological, and metabolic pathways (21, 22) (Figure 1).

Gut microbes actively produce a variety of bioactive compounds, including short-chain fatty acids (SCFAs) and essential vitamins. Many of these compounds enter the bloodstream, where they influence gene expression and play a key role in regulating immune function and metabolic activity (25, 26). The gut microbiota plays a crucial role in producing specific vitamins, such as vitamin K and B-complex vitamins, transforming bile acids, and metabolizing xenobiotics (27). These functions highlight its metabolic versatility and reliance on host physiological systems. The gut microbiota also plays an important role in the immune system by enabling it to distinguish between beneficial and pathogenic microbes and by inducing immune tolerance via pattern recognition receptors, including Toll-like receptors (TLRs) and NOD-like receptors (28, 29). However, growing evidence suggests that the physiological effects of these metabolites depend on both their concentrations and the overall microbial ecological context. This highlights that functional dysbiosis may occur even without major taxonomic alterations.

Disturbances in the balanced relationship between a host and their gut microbiota correlate with a broad spectrum of health conditions, such as IBDs, obesity, type 2 diabetes, cardiovascular diseases, and neurodegenerative disorders (30, 31). Whether microbial alterations primarily drive disease or result from host pathology remains unresolved for many chronic disorders. This distinction is important when evaluating microbiota-targeted nutritional interventions, including BAPs, because therapeutic efficacy may vary depending on disease stage, baseline microbiota composition, and host factors. More recent studies have demonstrated the existence of a two-way communication between the gut microbiota and the central nervous system, as the microbiota-gut-brain axis. This interaction affects mood, cognition, and behaviors by acting on microbial metabolites, vagal signaling, and regulation of neuroinflammatory pathways (32, 33). Also, various microbial strains can interact with dietary peptides via proteolysis or bioconversion, generating new fragments with enhanced immunomodulatory, antihypertensive, and antioxidative capacities (34). This mutualism not only enhances the therapeutic potential of dietary interventions but also shows the co-evolution of humans and their microbiota (35, 36). Therefore, a deeper understanding of host–microbiota–peptide interactions is essential to advancing precision nutrition strategies and microbiota-targeted therapies.

## 3 Bioactive peptides: an overview

**Figure 2:**
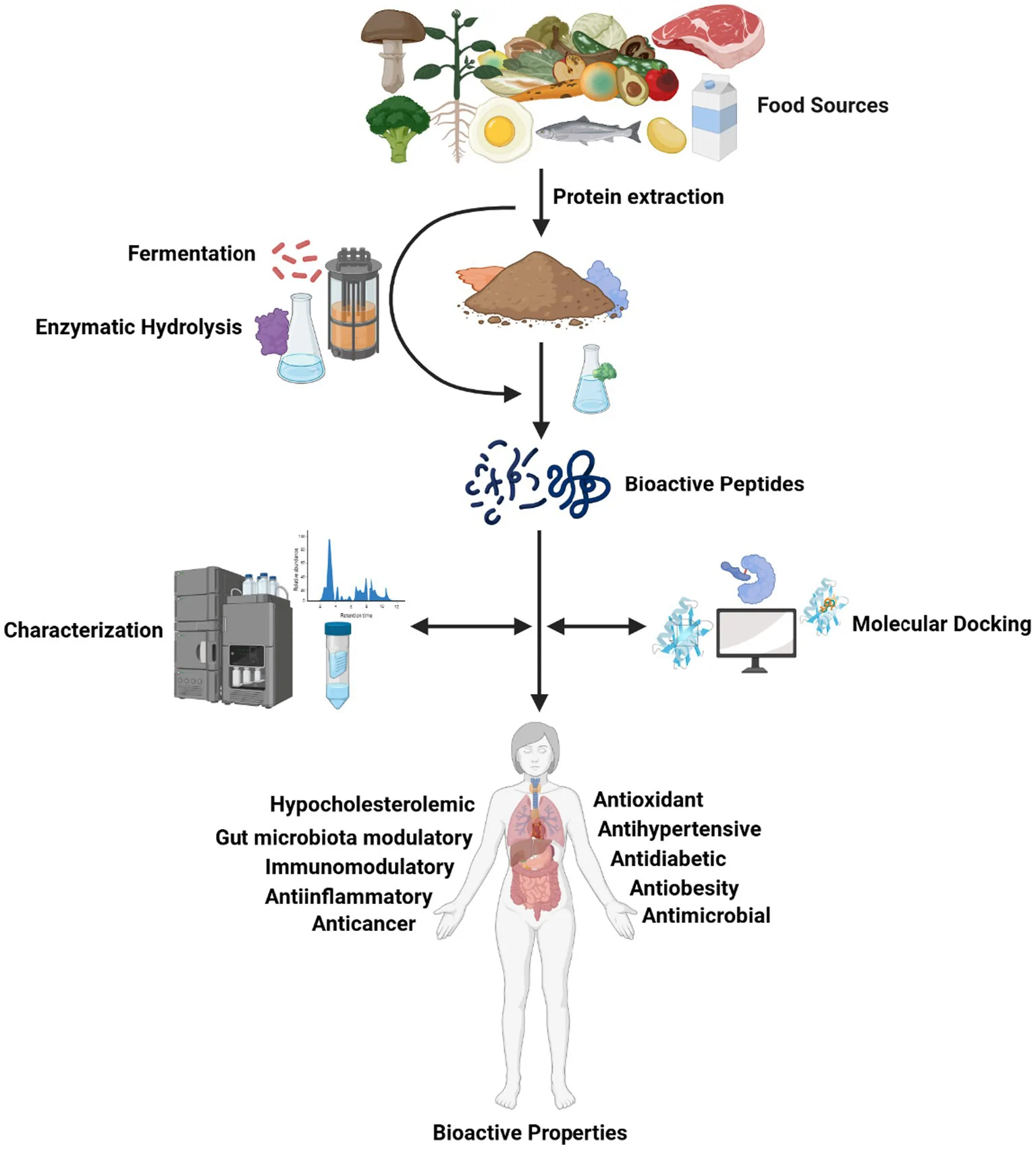
Overview of the production, characterization, and biological activities of food-derived bioactive peptides. Proteins extracted from plant- and animal-based food sources are converted into bioactive peptides through enzymatic hydrolysis and/or microbial fermentation. The resulting peptides are characterized using advanced analytical techniques and evaluated by molecular docking to predict peptide–target interactions. Food-derived BAPs exhibit diverse biological activities, including antioxidant, antihypertensive, antidiabetic, anti-obesity, antimicrobial, hypocholesterolemic, gut microbiota-modulatory, immunomodulatory, anti-inflammatory, and anticancer effects, highlighting their potential applications in functional foods, nutraceuticals, and therapeutics.

Specific protein fragments that perform fundamental physiological functions beyond serving as a source of nutrition are referred to as BAPs. Although these peptides are embedded in parent proteins in an inactive form, they are released and become biologically active through enzymatic hydrolysis or gastrointestinal digestion, or fermentation. The principal sources of BAPs are animal proteins (milk proteins, eggs, meat, and fish), plant proteins (soy, legumes, cereals, oilseeds, and others), and marine sources (algae and mollusks). Dairy peptides, particularly those derived from casein and whey, are among the most extensively studied due to their high bioavailability and diverse biological activities, including immunomodulatory, antihypertensive, antioxidant, and antimicrobial effects (6, 37–39). Understanding the sources, release mechanisms, and biological functions of BAPs provides a foundation for exploring their therapeutic potential in health and disease. As illustrated in Figure 2, proteins are first extracted from food sources and then hydrolyzed enzymatically or by microbial fermentation to release BAPs. Enzymatic hydrolysis is widely preferred because it enables controlled protein hydrolysis, yielding peptides with specific functional and therapeutic properties. The resulting peptides are purified and characterized using advanced analytical techniques, such as mass spectrometry, to determine their molecular weight, amino acid sequence, and structural properties (37, 40). In addition, molecular docking approaches are increasingly used to predict peptide–target interactions and evaluate their biological potential. These BAPs exhibit diverse health-promoting activities, including antioxidant, antimicrobial, antihypertensive, antidiabetic, cardioprotective, and gut health–modulating effects (39, 41).

BAPs diverge in length and sequence, hydrophobicity, net charge, and amino acid composition, and all of these are crucial to their respective functions and interactions with microbial communities. Hydrophobic and positively charged dipeptide-containing peptides tend to have greater antimicrobial and immunomodulatory effects, usually due to their interactions with microbial membranes or their regulation of host cellular pathways that control microbiota composition (42, 43). Some BAPs act as prebiotics, selectively promoting the growth of beneficial bacteria, including *Lactobacillus* and *Bifidobacterium*, and reducing the growth of opportunistic pathogens through antimicrobial activity or quorum-sensing inhibition. Moreover, certain peptides, including proline and branched-chain amino acids, are not susceptible to proteolytic breakdown and can enter the colon in their original form, thereby affecting the ecology of microorganisms (9, 44, 45).

The biofunctional properties of BAPs are commonly achieved through direct contact with microbial metabolites or by altering gut barrier activity, mucosal immunity, and microbial communication pathways. It is the synergistic combination of their disparate functions, natural sources, and low toxicity that makes BAPs promising for the creation of future-generation functional foods and nutraceuticals aimed at regulating the gut microbiota (46). Recent developments in peptide sequencing, computational prediction, and bioinformatics have accelerated the identification and characterization of novel BAPs with high affinity for gut-related targets. Their origins, sequences, and physicochemical characteristics shape the complex structures and functions of BAPs, making them potential regulators of the gut microbiota-host relationship (39, 41). This makes them the leaders of nutritional therapeutics, in which individualized diets can be designed to enhance microbial balance and treat chronic metabolic and inflammatory disorders.

## 4 Interaction between bioactive peptides and gut microbiota

**Figure 3:**
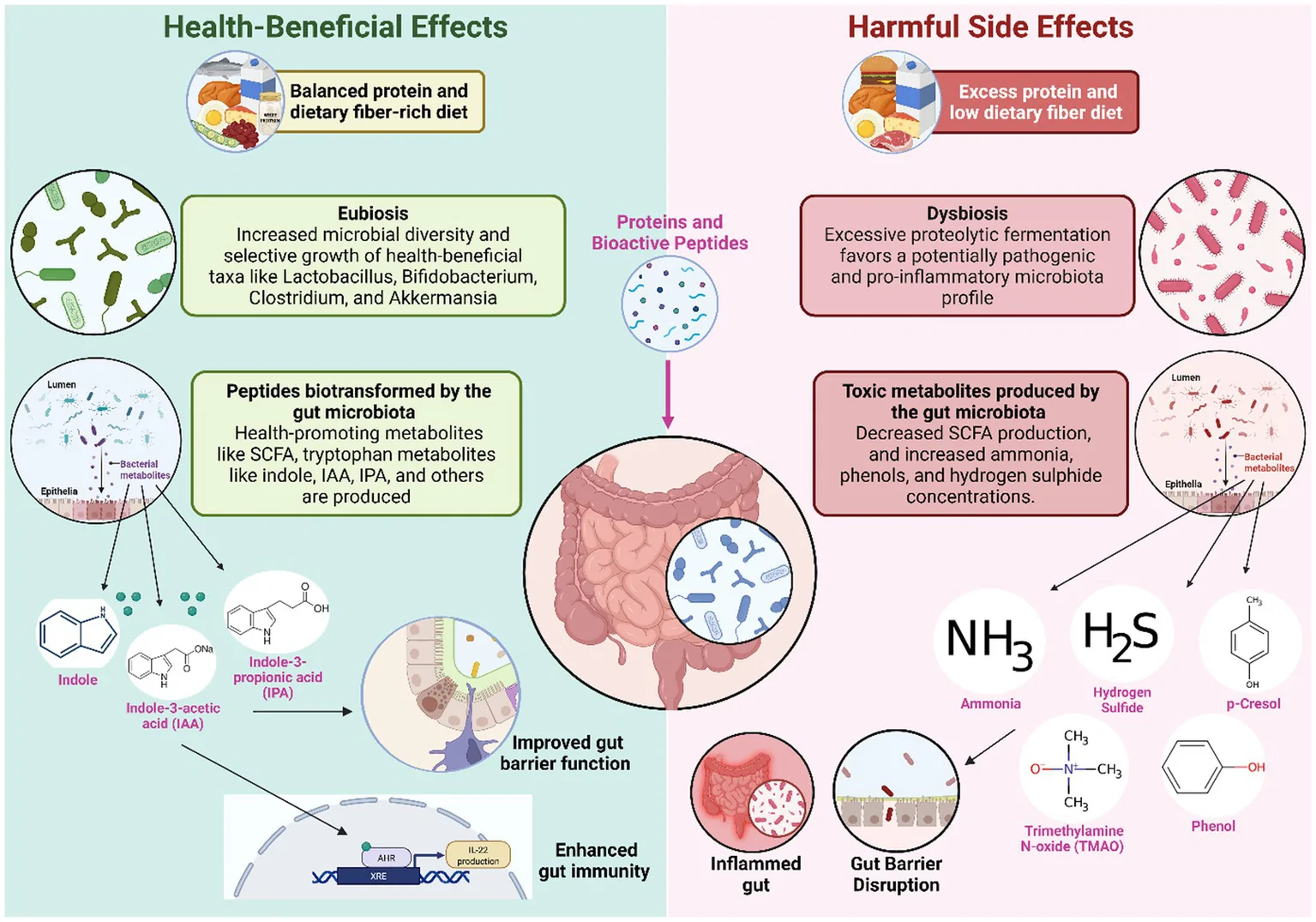
Effects of dietary protein and bioactive peptides on the gut microbiota and host gut health under balanced vs. imbalanced dietary conditions. In the context of a balanced, protein and dietary fiber-rich diet, microbial fermentation of proteins and bioactive peptides promotes eubiosis, characterized by increased microbial diversity and the selective enrichment of beneficial taxa (e.g., *Lactobacillus*, *Bifidobacterium*, *Clostridium*, and *Akkermansia*). Microbiota-mediated biotransformation of peptides yields health-promoting metabolites, including SCFAs and tryptophan-derived indole metabolites such as indole, indole-3-acetic acid (IAA), and indole-3-propionic acid (IPA). These metabolites strengthen intestinal barrier integrity and enhance mucosal immunity, in part through aryl hydrocarbon receptor (AhR)-mediated signaling at the xenobiotic response element (XRE) and the consequent induction of IL-22 production. On the other hand, an excess-protein, low-fiber diet causes excessive proteolytic fermentation. It leads to dysbiosis, favoring a potentially pathogenic and pro-inflammatory microbial community. This shift is accompanied by decreased SCFA production and increased generation of putrefactive and potentially toxic metabolites, including ammonia (NH_3_), hydrogen sulfide (H_2_S), phenol, *p*-cresol, and trimethylamine N-oxide (TMAO). Accumulation of these metabolites contributes to intestinal inflammation and gut barrier disruption.

The gastrointestinal tract is not only the main site of nutrient digestion and absorption but also a dynamic interface where dietary components continuously interact with the gut microbiota. Among these components, BAPs have gained attention for their bidirectional interactions with intestinal microorganisms (47). BAPs can act as signaling molecules, antimicrobial agents, enzyme substrates, and modulators of microbial metabolism (48, 49). This multifaceted role sets BAPs apart from other dietary bioactives and suggests their physiological effects go beyond simple nutrition. These interactions associated with different levels of human health, such as immune function, metabolism, and mental health. There is mounting evidence that endocrine regulatory or signaling molecules are generated by the microbial fermentation of dietary substrates. These metabolites play a crucial role in human health, as they enter the bloodstream and facilitate communication between the host and the microbiota (50, 51) (Figure 3).

Historically, research on diet–microbiota interactions has focused mainly on dietary fiber and other fermentable carbohydrates because of their established role in SCFA production. Only in the past decade has evidence suggested that specific peptides can selectively modulate microbial composition, epithelial barrier integrity, immune signaling, and microbial metabolite production (12, 52). Transport of BAPs within the intestine occurs rapidly; the intestinal epithelial layer is covered with mucus and consists of absorptive cells designed to block macromolecules, antigens, and microbial cells. Nonetheless, research indicates that peptides can bypass intestinal motility, influence gut immunity and physiology, and elicit systemic effects. Peptide absorption occurs through several mechanisms, including paracellular transport via tight junctions (TJ), passive diffusion, endocytosis, and carrier-mediated transport systems such as the peptide transporter 1 (PepT1), the major intestinal di- and tripeptide transporter in humans (48).

Following intestinal digestion, many peptides undergo further biotransformation by microbial proteases and peptidases in the colon. This process generates smaller peptide fragments and metabolites with altered biological activities. Several bacterial genera, including *Lactobacillus*, *Bifidobacterium*, and certain *Clostridium* species, have proteolytic systems capable of peptide metabolism. BAPs can be microbially degraded to reduce peptide-related toxicity, as seen with microbial degradation of gluten-derived peptides in relation to celiac disease (47, 53). Thus, the microbiota are transformers and modulators of peptide function. However, whether the observed biological activities come from the original dietary peptides or microbiota-derived metabolites has rarely been investigated. Recent studies indicate that BAPs have the potential to enhance probiotic growth and reduce the risk of metabolic disorders. BAPs from albumin, soybean, and egg white have been reported to profoundly affect the composition and activity of the gut microbiota (54). Studies have shown that peptides derived from milk, soy, fish, and other sources can selectively stimulate the growth of beneficial bacteria and suppress pathogenic strains (46). For instance, lactoferricin, a lactoferrin-derived peptide, exhibits antimicrobial activity against several Gram-positive and Gram-negative bacteria, potentially helping maintain a balanced microbial environment (55). Moreover, the presence of BAPs as prebiotics has been shown to increase the abundance of commensal species, including *Akkermansia muciniphila* and *Faecalibacterium prausnitzii*, which are known to support mucosal barrier health and the production of anti-inflammatory metabolites (56). The implications of these effects suggest that targeted microbiota-based nutraceutical development using specific peptides may be possible. However, considerable variability exists among studies regarding the magnitude of microbial changes, likely reflecting differences in peptide dose, treatment duration, host physiology, and baseline microbiota composition. These observations suggest that peptide efficacy is highly context-dependent rather than universally applicable.

One of the most consistently reported consequences of peptide-induced microbiota modulation is increased production of SCFAs, particularly acetate, propionate, and butyrate. These microbial metabolites support epithelial barrier maintenance, immune regulation, and host metabolic homeostasis. Several BAPs have been reported to stimulate the proliferation of bacteria that produce SCFAs, thereby establishing a positive feedback mechanism that promotes gut and overall health (57–59). Microbial products such as indoles, phenolic acids, and tryptophan analogs can also modify the expression of peptide transporters in the gut lining and, consequently, the absorption and systemic distribution of BAPs (60, 61). This biochemical communication indicates an active, complex interaction that affects the host’s physiological environment. However, establishing a direct causal relationship between peptide intake, microbial changes, SCFA production, and clinical outcomes remains challenging because few studies quantify all components of this pathway simultaneously.

The growing understanding of peptide–microbiota interactions has generated interest in developing microbiota-targeted nutritional interventions for chronic diseases such as IBDs, obesity, metabolic syndrome, and neurodegenerative disorders (62–64). For example, experimental models of colitis have shown that egg-derived peptides have anti-inflammatory and gut-protective properties, in part by altering the gut microbiota (65). These results support the significance of the use of BAPs in individualized nutrition regimens to address microbial imbalance and improve disease outcomes. Additionally, BAPs are being tested alongside prebiotics, probiotics, or polyphenols to enhance their gut-modulating effects. A promising approach is to design probiotic strains that synthesize specific BAPs in the gut, thereby transforming the microbiota into a biological bioreactor for therapeutic peptide production (66). Although the therapeutic potential of microbiota-targeted peptides is encouraging, robust clinical validation remains a priority.

While BAPs and high-protein dietary interventions are increasingly recognized for their therapeutic and functional benefits, excessive protein intake, combined with reduced dietary fiber, may adversely affect the gut microbial ecology (Figure 3) (67). Such dietary patterns can shift the colonic microbiota toward a proteolytic, potentially pathogenic composition, marked by enrichment of pro-inflammatory bacterial taxa (68). In the absence of sufficient fermentable fiber, microbial metabolism preferentially uses undigested proteins and peptides, generating harmful metabolites, including ammonia, phenolic compounds, indoles, biogenic amines, and hydrogen sulfide (69). These metabolites have been linked to impaired intestinal barrier integrity, mucosal inflammation, oxidative stress, and increased susceptibility to gastrointestinal disorders (70). Furthermore, reduced fiber availability limits the production of beneficial SCFAs, particularly butyrate, which plays a critical role in maintaining colonic epithelial health and immune homeostasis (71). Therefore, although BAPs possess promising nutraceutical potential, their long-term effects on the gut microbiota require careful evaluation within the broader dietary context.

Despite growing evidence of interactions between BAPs and the gut microbiota, much of it comes from *in vitro* fermentation systems and animal models. These approaches provide valuable mechanistic insights but do not fully capture the complexity and individual variability of the human gut microbiome. Differences in peptide sources, purification methods, dosages, intervention durations, and microbiome analysis pipelines also make direct comparisons across studies challenging. Overall, while modulation of beneficial microbial taxa has been consistently reported, the magnitude and reproducibility of these effects in humans remain uncertain.

## 5 Gut microbiota-mediated health benefits of bioactive peptides

### 5.1 Anti-inflammatory and antimicrobial effects

Inflammation is a natural immune response that helps the body fight infections, repair damaged tissues, and promote healing. Inflammation can be acute or chronic, with the latter associated with diseases such as arthritis, cardiovascular disease, cancer, and obesity (72, 73). Thus, managing inflammation is an important health goal. While anti-inflammatory drugs are common, research indicates they can have side effects such as high blood pressure, kidney damage, heart problems, acute kidney injury, and gastrointestinal issues (74). Additionally, although antibiotics may be used to treat infection and inflammation, their use can also promote the development of antibiotic-resistant microorganisms. Increasing evidence suggests that food-derived BAPs can modulate inflammatory responses not only through direct regulation of host immune signaling but also indirectly by altering gut microbial composition and microbial metabolite production (Table 1). One of the most well studied mechanisms by which BAPs influence intestinal inflammation involves their interaction with the gut microbiota and host immune signaling pathways. This process occurs through a series of coordinated events. (1) Pathogen-associated molecular patterns (PAMPs), conserved molecular structures found on microorganisms, particularly pathogens, that are recognized by host pattern recognition receptors (PRRs), triggering innate immune responses and initiating host defense mechanism**s**. For example, LPS from Gram-negative bacteria, peptidoglycan from Gram-positive bacteria, bind to TLRs on intestinal epithelial cells (IECs) and Paneth cells. (2) Upon activation, TLRs signal through the adaptor protein MyD88, which leads the activation of IRAK kinases. (3) This leads to subsequent activation of IκB kinase (IKK) complex, followed by phosphorylation of IκB, and release of NF-κB. (4) NF-κB drives transcription of pro-inflammatory cytokines (TNF-*α*, IL-1*β*, IL-6) and of antimicrobial peptides including β-defensins (hBD-2, hBD-3), RegIIIα/*γ*, and LL-37. (5) In addition, TLR activation triggers basal AMP and interleukin-18 (IL-18) production through the activation of NF-κB and inflammasome pathways, respectively. Together, these responses help fine-tune immune tolerance toward the gut microbiota while preserving intestinal homeostasis (75, 76). Marine-derived peptides demonstrate a marked reduction in pro-inflammatory cytokine levels (TNF-*α*, IL-1β) and a favorable modulation of the gut microbiota, notably promoting the proliferation of beneficial genera such as *Lactobacillus* and *Lachnospiraceae*. Mechanistically, their actions entail the manipulation of critical signaling pathways, including NF-κB and TLR-4, as well as the regulation of innate and adaptive immune responses (77). Although, direct evidence showing peptide-induced modulation of the gut microbiota and changes in these pathways in humans remains limited. Therefore, the proposed mechanisms should be regarded as biologically plausible rather than conclusively established in clinical settings.

**Table 1:** Recent studies on bioactive peptides influencing gut microbial composition and host physiological responses.

Bioactive peptide source	Peptide sequence, peptide type, or hydrolysates	Primary microbiota-mediated activity	Molecular targets/pathways	Level of evidence	Experimental model	References
Soybean	Fermented soybean meal peptides	Prevents microbial translocation and improves epithelial integrity	Upregulation of tight junction proteins	Preclinical	*In vivo* and Animal model	(142)
Algal protein hydrolysate (*Chlamydomonas reinhardtii*)	EWRPF and other Peptide fractions	Inhibition of pathogenic *Salmonella* growth	Interaction with essential bacterial proteins	*In vitro*	*In vitro* and in silico analyses	(143)
Bovine casein	KNR50 peptide	Broad-spectrum antimicrobial, antibiofilm, antiviral, and immunomodulatory activities	Membrane disruption and biofilm inhibition	*In vitro*	*In vitro*	(79)
Soybean	7S globulin-derived peptides	Increase in the relative abundance of human gut microbiome (Lachnospiraceae and Lactobacillaceae)	Suppressed proinflammatory Gram-negative bacteria and promoted the highest production of short-chain fatty acids	*In vitro*	*In vitro*	(144)
Chickpea	Chickpea protein hydrolysate	Gut microbiota modulatory and antioxidant effect	Showed the highest antioxidant ability, promoted the growth of Bifidobacterium, Pediococcus, Weissella, and acetate/propionate-producing bacterium Veillonella, stimulated the generation of short-chain fatty acids (SCFAs), and suppressed the generation of byproducts, including ammonia and indole	*In vitro*	*In vitro* fermentation model	(145)
Koumiss (fermented mare’s milk)	MP-4 peptide	Antibacterial activity against pathogenic bacteria by suppressing pathogenic *Escherichia coli* colonization	Disruption of bacterial membrane integrity and modulation of host metabolism	Preclinical	Animal model	(78)
Walnut	YVLLPSPK	Neuroprotective and antioxidant activity	Activation of Nrf2/HO-1 pathway and inhibition of NF-κB/p38 MAPK	Preclinical	Mouse model	(146)
Fish	Fish protein hydrolysates	Improved growth performance, reduced larval deformity rate, and increased the activity of digestive enzymes	Upregulated the expression of intestinal LAT2 (PepT2) and LAT1	Preclinical	Larval largemouth bass (*Micropterus salmoides*)	(147)
Egg	Egg white protein hydrolysate	Prevent colonic histopathological damage	Decreased pro-inflammatory interleukin and TNF-α also improved the expression of tight junction proteins in the colon	Preclinical	Rats (Crohn’s disease)	(148)
Lupin	Lupin protein hydrolysates	Gut microbiota modulatory effect	Improved weight gain, adiposopathy, and gut dysbiosis	Preclinical	Mice (High-fat diet)	(149)
Rice	Rice bran protein hydrolysate	Attenuated oxidative stress and inflammatory responses in DSS-induced colitic mice	Reduced pro-inflammatory mediators, decreased serum concentrations of fatty acid-binding protein 2 (FABP2), diamine oxidase (DAO), and D-lactic acid (D-LA), and increased colonic IL-10 content	Preclinical	Mice (DSS-induced colitis)	(150)
Casein (milk)	Casein glycomacropeptide	Modulates gut microbiota and enhances short-chain fatty acid production	Regulation of intestinal immune homeostasis and SCFA metabolism	Clinical	Human clinical study	(151)
Casein (milk)	Bovine kappa-casein glycomacropeptide	Anti-adhesive, prebiotic, and immunomodulatory properties	Alterations in gut microbiota composition, fecal and blood inflammatory markers, and gut-related symptoms	Clinical	Human study	(152)

In a recent study, a novel koumiss-derived peptide (MP-4) demonstrated potent antibacterial activity against *Escherichia coli* and mitigated infection-induced intestinal damage by modulating membrane integrity, host metabolism, and gene expression (78). Furthermore, a bovine casein-derived cationic peptide (KNR50) exhibited broad-spectrum antimicrobial activity, as well as antibiofilm, antiviral, and immunomodulatory activities, highlighting its potential as a multifunctional agent for therapeutic and food-preservation applications (79). Cationic antimicrobial peptides (AMPs), both endogenous (like defensins) and dietary (like lactoferricin, caseicins, and ovotransferrin) physically interact with microbial cell membranes (80, 81). Their biological functions in innate immunity, their structure and activity relationships, along with their mechanisms of action and therapeutic potential, are of great interest. Long-standing interactions between the host defense system and microorganisms have established multiple mechanisms that ensure commensal or mutualistic co-existence in the gut. This means that endogenous AMPs do not indiscriminately kill all bacteria. Rather, they preferentially suppress Gram-negative pathogens while being relatively permissive to Gram-positive commensals like *Lactobacillus* and *Bifidobacterium* (82). Collectively, these findings underscore the immense potential of BAPs as safe, multifunctional, and effective alternatives for modulating inflammation, enhancing immune responses, and promoting overall health. Although these findings are promising, the studies mainly evaluated pathogen inhibition rather than full modulation of the gut microbial community. Additional studies using microbiome sequencing and functional analyses are needed to determine broader microbiota-modulating effects beyond antimicrobial activity.

### 5.2 Implications for gastrointestinal disorders

BAPs have emerged as promising candidates for the prevention and management of gastrointestinal disorders through their combined effects on gut microbiota composition, intestinal barrier function, immune regulation, and microbial metabolism. A substantial body of preclinical research indicates that BAPs exert indirect health effects by reshaping the structural composition and functional activity of the gut microbial ecosystem which is itself a primary determinant of intestinal homeostasis. Gut microbial dysbiosis is a common feature of gastrointestinal disorders, including IBDs, irritable bowel syndrome (IBS), and colorectal cancer (CRC). By restoring microbial balance, BAPs may help alleviate disease-associated intestinal dysfunction (83, 84). BAPs selectively stimulate the growth of beneficial commensal genera, particularly *Lactobacillus*, *Bifidobacterium*, and *Akkermansia muciniphila*, while suppressing pro-inflammatory pathobionts such as *Escherichia coli*, *Clostridium difficile*, and *Desulfovibrio* spp. (85–87). As a consequence, this remodeling leads to elevated colonic production of SCFAs, specifically butyrate, propionate, and acetate. These SCFAs serve as the primary energy substrate for colonocytes, activate GPR41/43 on enteroendocrine and immune cells to suppress pro-inflammatory cytokine production, and promote the differentiation of regulatory T cells (Tregs) in the lamina propria, thereby reinforcing mucosal immune tolerance mediators (12, 88). Although increased SCFAs production is a frequently proposed mechanism linking BAPs with improved gastrointestinal health, direct causal evidence remains limited. Many studies measure SCFAs concentrations alongside microbial composition, but few have shown that these metabolites directly mediate improvements in epithelial integrity or inflammatory status. Future studies integrating metabolomics with mechanistic intervention experiments are needed to establish causality.

Experimental studies further indicate that BAPs restore epithelial barrier integrity by increasing the expression of TJ proteins, including claudin-1, occludin, and ZO-1, thereby reducing intestinal permeability (84). Specific BAPs upregulate TJ proteins, thereby decreasing intestinal permeability, also known as “leaky gut,” which has been linked to systemic inflammatory responses and the progression of localized gastrointestinal disorders (77, 89). BAP-mediated restoration of epithelial TJ integrity represents one of the most mechanistically consistent findings in the literature. At the molecular level, BAPs upregulate TJ protein expression through at least two plausible ways: (i) activation of the PI3K/Akt pathway, which promotes epithelial cell survival and assembly of claudin/occludin complexes at the junctional membrane; and (ii) suppression of NF-κB-mediated claudin-2 upregulation as claudin-2 forms leaky, cation-selective channels that are pathologically overexpressed in IBDs and markedly increase paracellular permeability. For instance, Bovine lactoferrin has been specifically shown to upregulate claudin-1, occludin, and ZO-1 expression at both the mRNA and protein level in Caco-2 cell monolayers and human intestinal epithelial cell models (90). Restoration of TJ proteins has been consistently observed in several experimental models, but it remains unclear if these molecular changes alone can produce sustained clinical remission in patients with IBDs. Longitudinal human studies that simultaneously examine barrier function, microbiota dynamics, and clinical outcomes are still lacking.

Recent findings suggest that food-derived BAPs, including those from milk, soy, and wheat, may help regulate carcinogen production by influencing the gut microbiome, potentially reducing the risk of colorectal cancer (CRC). Their antioxidant properties also protect against DNA damage and carcinogenic transformation in the colonic epithelium (91). BAP-driven enrichment of SCFAs-producing taxa elevates colonic butyrate concentrations. Butyrate acts as a Histone deacetylases (HDACs) inhibitor and it promotes tumor suppressor gene expression and induces cell cycle arrest and apoptosis in cancerous colonocytes. It also suppresses Akt phosphorylation (counteracting PI3K-Akt-NF-κB-mediated pro-tumorigenic signaling driven by pathogens like *Fusobacterium nucleatum*). It inhibits aerobic glycolysis in cancer cells via GPR109a signaling by reducing GLUT1 surface expression and impairing G6PD activity (92). However, the current evidence is mainly based on experimental animal models or mechanistic cell culture systems and has not been validated in clinical settings.

The microbiota-modulating effects of peptides can also regulate bile acid metabolism, facilitate lipid digestion, and reduce gastrointestinal dysfunctions associated with bile. Bile acids are synthesized from cholesterol in the liver as primary bile acids [cholic acid (CA) and chenodeoxycholic acid (CDCA)] and biotransformed by Bile salt hydrolase (BSH)-expressing gut bacteria to secondary bile acids [deoxycholic acid (DCA) and lithocholic acid (LCA)]. They function not merely as lipid emulsifiers but as potent signaling molecules. They activate two key intestinal epithelial receptors: the nuclear farnesoid X receptor (FXR) and the membrane-bound G-protein-coupled receptor TGR5 (93). Gut microbiota regulates the ratio of primary to secondary bile acids and the enterohepatic circulation of bile acids. Using more advanced applications, peptides can be combined with probiotics or prebiotics, so-called synbiotics, to have synergistic effects that can be used to prevent and treat complex and chronic gastrointestinal diseases (94, 95). Despite strong preclinical evidence and mechanistic understanding, significant gaps remain in research, especially in demonstrating clinical efficacy, identifying optimal peptide formulations and dosages, and establishing safety in humans. Overall, evidence indicates that BAPs have considerable potential to improve gastrointestinal health through coordinated effects on gut microbiota, epithelial barrier function, immune regulation, and microbial metabolism. However, substantial knowledge gaps remain about optimal peptide selection, dose–response relationships, long-term safety, and individual variability in treatment response.

### 5.3 Neuroprotective and gut-brain axis modulation

BAPs have emerged as potent mediators of neuroprotection and modulation of the gut-brain axis, with significant implications for nutritional therapeutics and personalized nutrition. Increasing evidence highlights the diverse roles these peptides play, ranging from directly protecting neurons to regulating communication between the central and enteric nervous systems via the gut microbiota axis. Their neuroprotective actions primarily involve reducing neuronal inflammation, preventing apoptosis, and managing oxidative stress, laying the groundwork for interventions in neurodegenerative diseases (96–98). The mechanisms underlying the neuroprotective effects of BAPs are not fully understood. While some low-molecular-weight peptides may cross the blood–brain barrier (BBB) via carrier-mediated transport or transcytosis and exert direct neuroprotective effects, evidence of BBB permeability of most BAPs remains limited (99). Several food-derived BAPs have shown neuroprotective properties in experimental models by reducing oxidative stress, suppressing neuroinflammation, and limiting neuronal apoptosis. Nevertheless, few studies have simultaneously examined changes in gut microbial composition, microbial metabolites, and neurological outcomes. Peptides derived from sources such as walnut proteins (e.g., LPF, GVYY, WEKPPVSH) have demonstrated significant efficacy in alleviating memory deficits in animal models by modulating microglial activity, attenuating neuroinflammatory cascades, and activating antioxidant defense pathways, including Nrf2/HO-1. These peptides support neuronal survival by inhibiting the NF-κB and p38 MAPK pathways and boosting antioxidant enzyme activities, ultimately decreasing oxidative damage in neural tissues (11, 100). Current evidence supports the hypothesis that BAPs help regulate the microbiota–gut–brain axis by influencing microbial metabolism, intestinal barrier function, immune signaling, and neuronal homeostasis. However, improving understanding of their specific microbiota interactions will be essential to unlocking their clinical potential to manage neurodegenerative diseases and enhance cognitive health through personalized nutrition. Future studies integrating microbiome sequencing, metabolomics, and neurobehavioral assessments will be essential to clarify these complex interactions.

### 5.4 Role in metabolic regulation

BAPs demonstrate significant potential to regulate metabolism and counter the pathophysiology of obesity and type 2 diabetes through multiple, often interconnected mechanisms, with gut microbiota functioning as an active component (54, 101). BAPs help manage obesity by targeting key regulatory pathways in adipogenesis, lipid metabolism, appetite control, and gut-derived inflammatory signaling (102). BAPs primarily suppress the activity of peroxisome proliferator-activated receptor gamma (PPARγ), a ligand-activated type II nuclear receptor that functions as a key transcriptional regulator of adipogenesis, lipid storage, and glucose metabolism. By inhibiting PPARγ and its downstream transcriptional network, BAPs reduce the differentiation of preadipocytes into mature adipocytes and consequently limit intracellular lipid droplet accumulation. Concurrently, many BAPs upregulate PPARα expression in the liver and skeletal muscle, promoting fatty acid *β*-oxidation and lipolysis. This dual PPAR regulation, i.e., suppressing anabolic PPARγ while activating catabolic PPARα, is further reinforced through AMP-activated protein kinase (AMPK) activation, which phosphorylates and inhibits key lipogenic enzymes downstream of PPARγ. Moreover, BAPs likely suppress key lipogenic enzymes, including fatty acid synthase (FAS), lipoprotein lipase (LPL), and acetyl-CoA carboxylase (ACC), while simultaneously activating hormone-sensitive lipase (HSL), which is the rate-limiting enzyme in lipolysis (101, 103–105). Therefore, they create a metabolic environment unfavorable to fat accumulation and exert anti-obesity effect.

**Figure 4:**
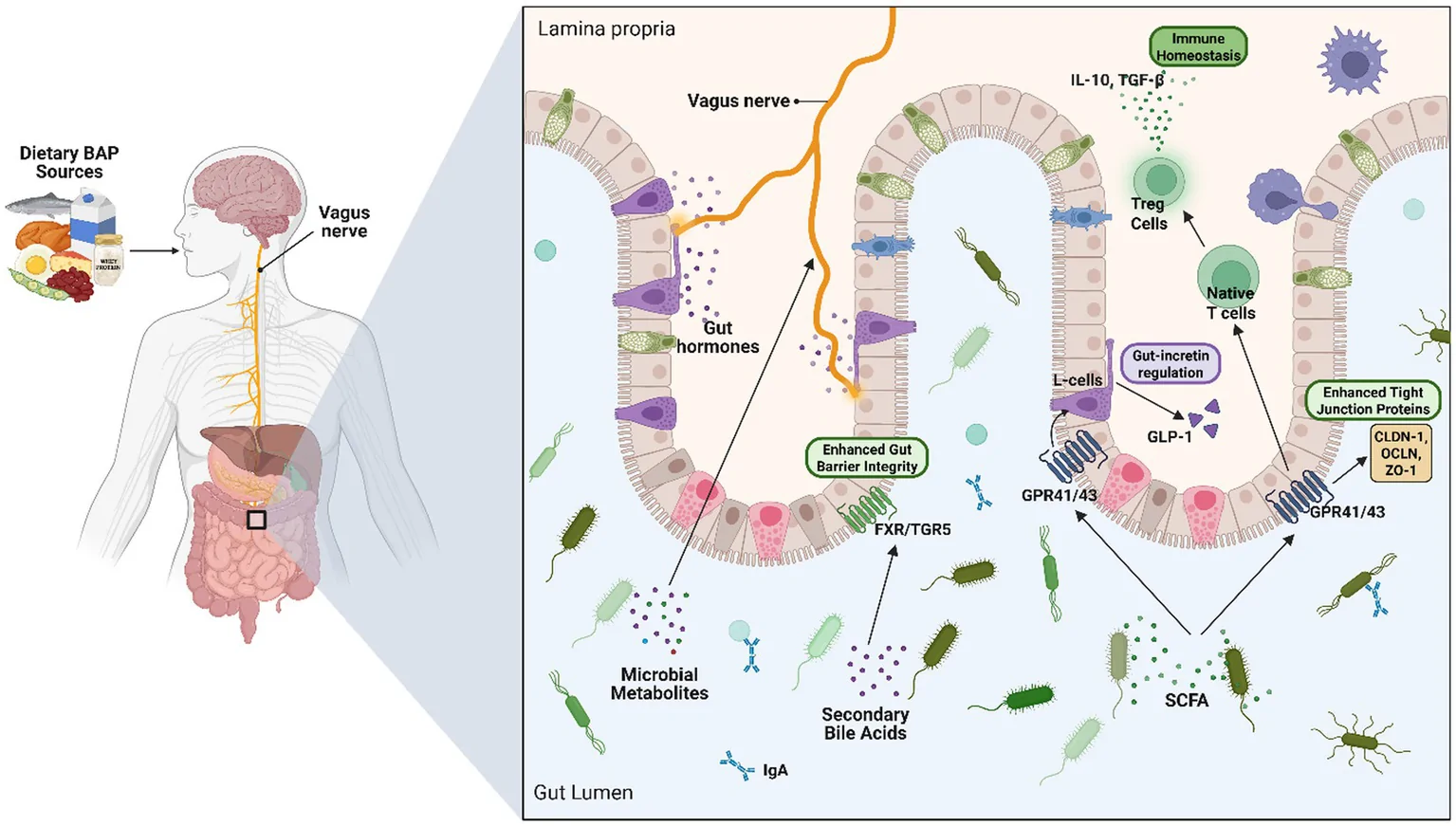
Mechanisms underlying dietary bioactive peptide-driven gut microbiota interactions in the regulation of the gut-X axis. Schematic representation of the molecular and cellular pathways through which BAPs and microbiota-derived metabolites modulate host physiology at the intestinal mucosa. Gut microbiota produced SCFAs engage the epithelial G protein-coupled receptors GPR41/43 (FFAR3/FFAR2), thereby (i) stimulating enteroendocrine L-cells to secrete the incretin hormone GLP-1 (gut-incretin regulation), (ii) promoting regulatory T-cell (Treg) differentiation from naïve T cells and the release of anti-inflammatory cytokines (IL-10, TGF-β) to support immune homeostasis, and (iii) enhancing the expression of TJ proteins (CLDN-1, OCLN, ZO-1) to reinforce epithelial barrier integrity. Secondary bile acids generated by microbial biotransformation signal through the FXR and TGR5 receptors to further enhance gut barrier integrity. The microbiota influences brain signaling through the vagus nerve to alter central neurotransmitter receptors. Secretory IgA within the lumen contributes to microbial regulation and mucosal immune surveillance. Note that these pathways are complementary and interconnected, but the causal mechanistic evidence comes almost entirely from germ-free and gnotobiotic rodent models, so human relevance remains largely correlational and not yet firmly established.

One of the most intriguing emerging mechanisms is the ability of BAPs to indirectly influence appetite regulation by modulating microbial metabolite production. SCFAs generated by gut microorganisms activate host cell receptors GPR41 and GPR43 on enteroendocrine L-cells, thereby stimulating secretion of GLP-1 and peptide YY (Figure 4) (106). BAPs are also reported to play a role in the suppression of chronic inflammation in adipose tissue and the gut, reducing obesity-induced inflammatory signaling cascades and enhancing metabolic resilience (107). Furthermore, BAPs positively influence the gut microbiota by promoting the growth of beneficial bacteria, reducing the presence of pro-obesity microbes, and enhancing resistance to gastrointestinal stress, thereby supporting overall metabolic health.

In experimental models of type-2 diabetes, BAPs showed antidiabetic effects through several interconnected pathways, including: (1) enhancing insulin sensitivity by stimulating glucose uptake and transport, especially via increased Glucose Transporter Type 4 (GLUT4) expression and activity in muscle and adipose tissue; (2) modulating incretin hormones (GLP-1, GIP), boosting their secretion and prolonging activity through inhibition of dipeptidyl peptidase-4 (DPP-4). These changes improve pancreatic β-cell function and regulate postprandial glycemia; (3) inhibiting digestive enzymes such as *α*-amylase and α-glucosidase, resulting in slower carbohydrate breakdown and reduced glycemic spikes; (4) reducing oxidative stress and inflammation by suppressing NF-κB, JNK, and p38 MAPK pathways, and activating Nrf2-mediated antioxidant responses; and (5) modulating insulin signaling pathways, facilitating PI3K/Akt activation and GLUT4 translocation, thereby supporting glucose homeostasis and preventing insulin resistance (108–110). Although these mechanisms have substantial preclinical evidence supporting them, their clinical importance remains uncertain.

BAPs particularly influence the metabolic landscape by interacting with the gut microbiota. They selectively promote the growth of bacteria that produce beneficial metabolites, thereby strengthening gut barrier integrity and influencing host metabolism, which improves body weight regulation and insulin sensitivity. Enrichment in SCFAs, secondary bile acids, and beneficial indole derivatives, while depletion of ammonia, TMAO precursors, and hydrogen sulfide, constitutes the primary mechanistic bridge between BAP intake and improved metabolic outcomes in peripheral tissues. Animal and early human studies highlight the therapeutic and preventive potential of BAPs as dietary supplements for metabolic syndrome and related conditions. Progress depends on expanding knowledge of their absorption, bioavailability, and the relationship between peptide structure and microbiota composition, which is essential for developing precision nutrition strategies (103, 111). Overall, BAPs offer multiple metabolic benefits, underscoring their importance for targeted nutritional interventions in the management of obesity, diabetes, and other metabolic health disorders.

Although many studies report beneficial effects of BAPs on gut microbial composition and host physiology, not all show consistent outcomes. The extent of microbiota modulation varies depending on peptide structure, dietary background, disease status, and baseline microbial composition. In some studies, improvements in host physiology occurred without major changes in microbial diversity, suggesting direct host signaling pathways may also contribute to the benefits. These discrepancies highlight the need for standardized protocols and mechanistic studies to distinguish microbiota-dependent from microbiota-independent effects.

## 6 Technological approaches to enhance peptide-microbiota interaction

Although several BAPs have shown promising biological activities *in vitro*, their translation into functional foods and therapies is limited by poor gastrointestinal stability, low bioavailability, rapid enzymatic degradation, and insufficient targeting of the distal intestine. Consequently, research has focused on developing delivery technologies that preserve peptide integrity and maximize interactions with the gut microbiota. These advances form a critical bridge between peptide discovery and clinical application (112, 113). The primary challenges for food-derived BAPs are rapid degradation in the digestive tract, poor permeability, and limited bioavailability. Advanced delivery systems, such as encapsulation and nanoformulation, aim to address these issues. Encapsulation techniques involve encapsulating peptides within carriers, such as liposomes or hydrogels, that protect them from enzymatic degradation during gastrointestinal transit. Polymeric nanoparticles made from biocompatible polymers, such as poly(ethylene glycol)-block-polycaprolactone (PEG-PCL), help extend systemic circulation and improve stability by creating hydrophilic barriers and providing steric protection. Encapsulation also enables precise control of release, targeted delivery, and protection against environmental stressors (114). Despite these advantages, encapsulation technologies have several practical limitations. These include high production costs, limited scalability, potential interactions with food matrices, and uncertain long-term stability during storage. Furthermore, many encapsulation systems show promising performance only under laboratory conditions. Evidence from commercial food applications and human clinical studies remains scarce.

Nanotechnology offers platforms, such as liposomes, polymeric nanoparticles, and solid lipid nanoparticles, that can protect peptides from enzymatic breakdown and enable sustained release. Surface modification (e.g., ligand-based targeting) and stimuli-responsive systems further enhance delivery precision. Additionally, nanoformulation increases the chances of peptide interaction with specific gut microbes by promoting retention within targeted regions of the gastrointestinal tract (115–117). These carriers support peptide transport across the intestinal barrier and enhance uptake by microbiota populations residing in various gut niches. Peptide–drug conjugates and self-assembled peptide nanomaterials further expand the range of delivery methods, enabling improved tissue penetration and enhanced bioactivity. Utilizing these technologies is crucial not only for clinical translation but also for expanding the therapeutic applications of antimicrobial peptides to food safety.

Furthermore, integrating BAPs with probiotics, prebiotics, and synbiotics presents a promising approach to enhance their beneficial effects on the gut microbiota (118). Probiotics can alter the gut environment and enhance the absorption and use of BAPs. Probiotic fermentation also produces additional peptides and metabolic byproducts that work in conjunction with the added peptides (119). Non-digestible food components selectively support beneficial microbes, promoting their growth and activity. These prebiotic components help create a gut environment that supports the proliferation of microbes capable of interacting with and utilizing peptides, thereby enhancing health benefits (113). Combining probiotics and prebiotics as a synbiotic creates synergistic effects, where prebiotics serve as substrates that enhance the viability and colonization of probiotics. Synbiotic formulations that deliver both microbes and substrates, with or without BAPs, enhance modulation of the intestinal microbiota, increase production of beneficial metabolites such as SCFAs, and inhibit pathogens (120). When BAPs are incorporated into synbiotic formulations, they can serve as additional substrates for microbial fermentation, induce shifts in microbiota populations, and contribute to health benefits, including immune modulation and pathogen suppression. Increased tolerance to intestinal environmental stresses and effective metabolic interactions further highlight the value of synbiotic-peptide combinations. Although synbiotic formulations offer opportunities to enhance peptide efficacy, their biological performance depends on multiple host-specific factors. These include baseline microbiota composition, dietary habits, gastrointestinal transit time, and microbial metabolic capacity. Consequently, the effectiveness of combined peptide–probiotic formulations may vary substantially among individuals, highlighting the importance of personalized nutrition approaches.

Another important challenge is the lack of standardized methods to evaluate delivery efficiency. Most studies assess peptide stability using simulated gastrointestinal digestion models that cannot fully reproduce the complexity of the human gastrointestinal tract (121). Few investigations quantify the proportion of intact peptides reaching the colon (122) or directly correlate delivery efficiency with microbiota modulation and clinical outcomes. Developing standardized *in vitro–in vivo* correlation models is essential to compare different delivery platforms and optimize their therapeutic performance. From a translational perspective, technological feasibility should be evaluated alongside biological efficacy. Factors such as manufacturing costs, scalability, regulatory approval, consumer acceptance, storage stability, and compatibility with existing food-processing systems will determine whether peptide-based delivery technologies can be successfully commercialized. Recent technological advances have substantially improved opportunities for enhancing peptide stability, targeted delivery, and microbiota modulation. Nevertheless, most of these technologies remain at the proof-of-concept stage. Future research should prioritize clinically validated delivery systems that combine high encapsulation efficiency, scalable manufacturing, regulatory compliance, and demonstrable improvements in human health outcomes.

## 7 Translational challenges

**Figure 5:**
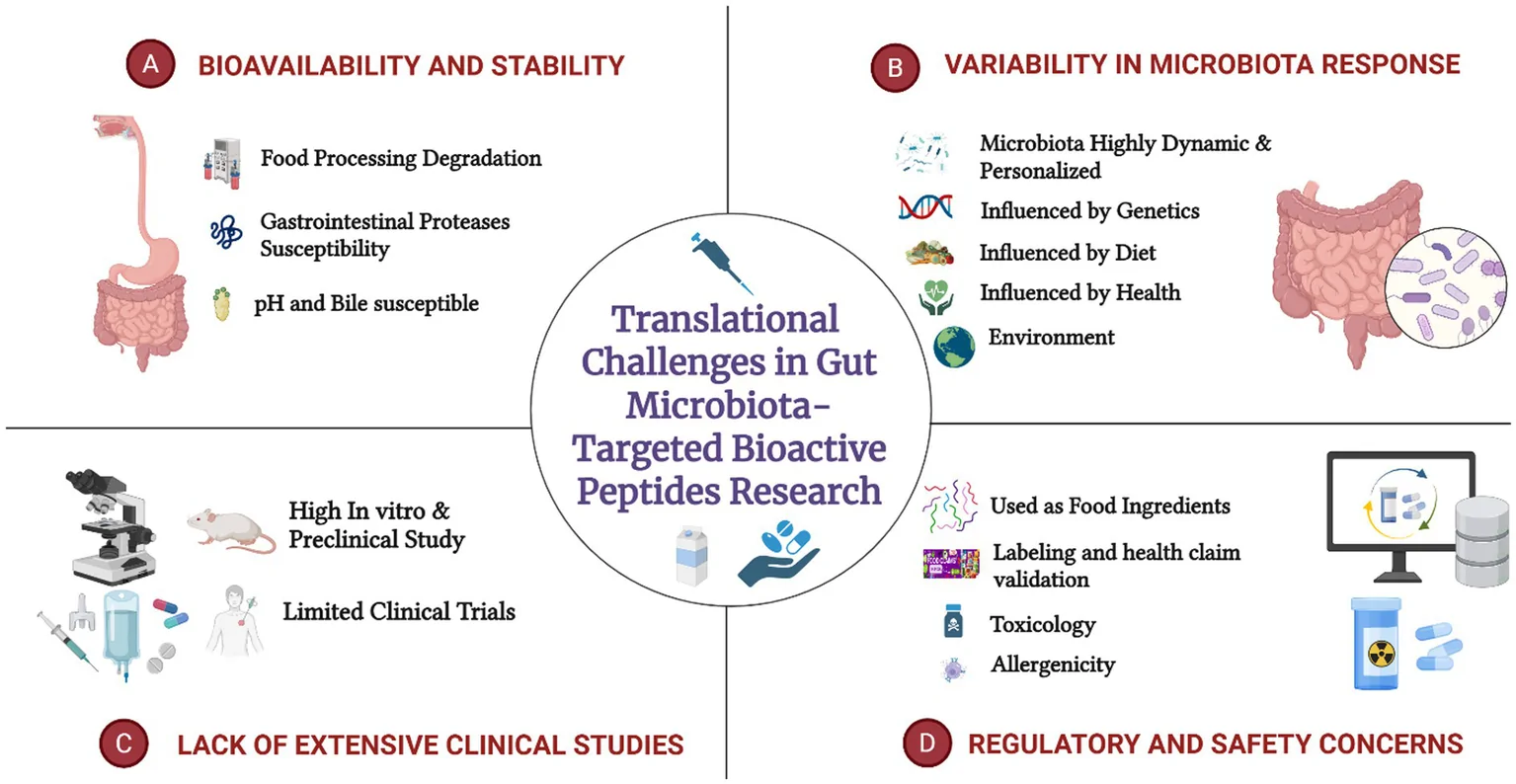
Major translational challenges limiting the clinical and commercial application of gut microbiota-targeted bioactive peptides. The principal barriers include: **(A)** Limited bioavailability and stability due to food processing, gastrointestinal proteolysis, and pH/bile sensitivity; **(B)** interindividual variability in gut microbiota composition and response, influenced by genetics, diet, health status, and environmental factors; **(C)** insufficient clinical evidence, with most studies remaining at the *in vitro* and preclinical stages; and **(D)** regulatory and safety concerns, including toxicity, allergenicity, labeling, health claim validation, and regulatory approval. Collectively, these challenges must be addressed to facilitate the successful translation of microbiota-targeted bioactive peptides into functional foods, nutraceuticals, and therapeutic applications.

Despite significant advances in gut microbiota-targeted BAPs, several major obstacles hinder their effective transition from laboratory research to mainstream nutritional therapies. Four primary challenges, bioavailability and stability issues, individual variability in microbiota response, lack of extensive human clinical studies, and regulatory and safety concerns, are particularly critical (Figure 5). Addressing and resolving these issues are essential steps to successfully adopt BAPs as a new approach to nutritional therapies.

One of the main challenges is the limited bioavailability and poor stability of food-derived peptides after ingestion. These peptides are particularly susceptible to degradation by gastrointestinal proteases, leading to the loss of their desired bioactivities before they reach the colon, where essential interactions with the gut microbiota occur. Additionally, the harsh pH, presence of bile salts, and the activity of endogenous and microbial enzymes in the gastrointestinal tract increase their instability, leading to the breakdown of these compounds into inactive fragments. Peptides can also decompose due to physical and chemical factors during food processing, storage, or transportation, making it even more challenging to deliver intact peptides to their functional site (115, 123, 124). The adoption of encapsulation technologies, such as liposomes, biopolymer nanoparticles, hydrogels, and emulsions, offers several advantages, including protection of peptides from digestion and enhanced targeted delivery to the colon. However, these methods often face challenges related to scalability, cost, and compatibility in real-world food systems. The risk remains that bioactive properties observed *in vitro* may not translate to *in vivo* conditions, primarily due to stability and bioavailability issues, as well as losses during digestion and metabolic transit (125).

Another major challenge is the significant individual variability in how microbiota respond to the same BAPs intervention. The human gut microbiome is highly dynamic and personal, influenced by genetics, diet, health, medications, and environmental factors. As a result, a peptide that alters microbiota composition or activity in one person may have no effect at all, or a very different effect, in another. This diversity is driven by differences in microbial substrate preferences, enzyme production, and metabolite output, which in turn affect how BAPs are metabolized and their subsequent effects. Recent metaproteomic studies show that not only are taxon-specific responses critical, but peptides can also cause unique, personalized changes in microbial abundance and function. This variability makes it challenging to develop standardized peptide-based treatments and complicates predicting their effectiveness across populations. New approaches that incorporate detailed microbiome profiling and personalized dietary plans are emerging to address these differences; however, these precision nutrition strategies remain under development and have not yet widely adopted in clinical practice (88, 126, 127).

Another major limitation is the lack of comprehensive, high-quality human clinical studies that specifically examine the effects of gut microbiota-targeted BAPs on health. Most of the current evidence supporting their benefits comes from *in vitro* fermentation models or preclinical animal experiments. While these are useful for understanding mechanisms, they cannot reliably predict human physiological responses. Human trials to date have often been limited by small sample sizes, short intervention durations, a variety of endpoints, and inadequate characterization of both peptide structures and microbiome changes. Standardized protocols for peptide delivery, dosage, and outcome measures are often lacking, and assessments of microbiota composition and function rarely integrate with host metabolic and immune responses. This evidence gap is exacerbated by challenges in achieving reproducible results due to individual differences and variations in baseline microbiome states. Without large, well-designed, randomized controlled trials that include long-term, multi-omics analyses, confirming the safety and effectiveness of BAPs therapies targeting the microbiota in humans remains difficult (11, 47, 100). Bridging this gap requires collaborative efforts across disciplines to design and conduct holistic clinical investigations.

Although most preclinical and clinical studies have evaluated BAPs as therapeutic interventions, their preventive potential through regular dietary intake also warrants consideration. Foods naturally rich in BAPs may help maintain gut microbial balance, intestinal barrier integrity, and immune homeostasis before disease onset. However, evidence supporting preventive effects in healthy populations remains limited, and long-term prospective and dietary intervention studies are needed to determine whether habitual consumption of BAP-rich foods can reduce the risk of chronic diseases by modulating the gut microbiota.

Regulatory and safety issues further complicate the development and use of gut microbiota-targeted BAPs. Unlike drugs, which face strict safety evaluations and regulations, BAPs used as food ingredients or supplements operate in a more uncertain regulatory environment, with rules varying across regions and countries. For example, peptides from allergenic foods such as dairy, soy, or wheat may pose a risk of allergic reactions or hypersensitivity. The potential for these peptides to cause unwanted immune responses or toxicity also needs evaluation. Additionally, the breakdown of certain peptides by gut microbes can produce harmful byproducts or promote the growth of nonbeneficial or pathogenic bacteria. This issue is exacerbated by the lack of standardized international guidelines for food-grade peptides, which cover aspects such as source documentation, processing, labeling, and health claim validation (12, 40, 84, 128). Comprehensive toxicological evaluations, allergenicity tests, and long-term safety assessments remain uncommon for most BAP products targeting the microbiota. Therefore, establishing unified, science-based regulatory guidelines and safety frameworks is essential to support responsible innovation in this sector and to safeguard consumer safety and trust.

Another important limitation is the considerable heterogeneity among existing studies. Differences in peptide preparation methods, molecular characterization, doses, intervention duration, sequencing platforms, and bioinformatic analyses reduce comparability and reproducibility of findings. Moreover, many studies rely on taxonomic changes without integrating functional analyses like metagenomics, metatranscriptomics, metabolomics, or host immune profiling (100). Future investigations should adopt standardized methodologies and multi-omics approaches to better establish causal relationships between BAPs, gut microbiota, and host health.

## 8 Conclusion and future research perspectives

Current evidence supports the potential of BAPs as modulators of gut microbial composition and host physiology. These peptides help maintain a balanced microbial environment, combat obesity, mediate immune functions, and promote healthy gut–brain communication. Their ability to selectively stimulate beneficial bacteria and suppress harmful species provides probiotic and prebiotic effects essential for gastrointestinal balance. Gut microbiota-targeted BAPs exhibit significant anti-inflammatory and immunomodulatory properties, influencing key signaling pathways and thereby reducing chronic inflammation associated with various diseases. However, most data come from experimental models, while evidence from well-designed human clinical trials remains limited. Understanding absorption and bioavailability, elucidating the exact mechanisms of host–microbiota interactions, and measuring health outcomes across diverse populations are crucial frontiers. Advances in omics technologies and personalized nutrition are expected to clarify these interactions, enabling tailored dietary interventions that harness the unique capabilities of gut microbiota-targeted BAPs.

The ongoing exploration of gut microbiota-targeted BAPs opens promising avenues for advancing nutritional therapeutics. Personalized nutrition and microbiota-focused peptide therapies represent a transformative shift away from “one-size-fits-all” dietary guidelines toward custom interventions tailored to each individual’s unique microbiome profile. Because individual microbiota composition significantly influences metabolic and immune responses to peptide intake, personalized nutrition models use baseline microbiome data to create peptide therapies that maximize health benefits for each person. The use of machine learning, computational modeling, and microbiome biomarker discovery enables the rational selection of peptide combinations and optimal delivery methods, accounting for variability in microbial composition, host genetics, and metabolic state. Recent studies have shown that predictive models can estimate individual glucose responses and metabolic improvements by linking dietary intake, such as fiber or peptides, with microbiome features, including the abundance of *Prevotella* and other genera. As large-scale population research and multi-center trials increase, a deeper understanding will develop regarding how food components interact with microbial functions across genetically and environmentally diverse groups, paving the way for personalized peptide-based nutrition strategies for disease prevention and treatment (129–132).

The integration of omics technologies provides unprecedented detail and accuracy for the research and development of gut microbiota-targeted peptide therapies. Metagenomics includes essential information on microbial genes and potential enzyme functions, while proteomics reveals changes in microbial and host protein expression in response to peptide treatments. Metabolomics tracks dynamic shifts in bioactive metabolites and metabolic pathways. These multi-omics approaches allow a systems-level understanding, connecting peptide intake to changes in microbial community structure, gene activity, protein functions, and host metabolism. For example, combining metagenomics and macroproteomics can reveal distinct protein expression patterns and metabolic capabilities in both healthy and diseased states. Meanwhile, metabolomic fingerprinting helps establish direct links between peptide therapy and improved host outcomes, including lower inflammation, reduced metabolic syndrome, and decreased cardiovascular risk. Advancing high-throughput sequencing, mass spectrometry, and computational bioinformatics will enhance the accuracy of these technologies, enabling more precise identification of BAPs, a deeper understanding of mechanisms, and real-time monitoring of physiological responses. Ultimately, this integrated, multi-layered analysis will be crucial for designing and clinically validating peptide therapies targeting the microbiota (133–137).

A third crucial future trend is the growing role of BAPs in functional foods and nutraceuticals. As awareness of their health benefits increases, food technology will focus on incorporating well-understood, microbiota-targeted peptides into mainstream products. Peptides with confirmed antihypertensive, antioxidant, immunomodulatory, and gut-barrier-protective effects can be extracted from various sources, including plants, marine organisms, dairy products, and fermented foods. Novel delivery methods, such as encapsulation or matrix embedding, enhance stability and enable targeted release in the colon, thereby boosting effectiveness and improving consumer convenience. The future of food BAPs is also influenced by increased regulatory activity, consumer interest in natural health-promoting ingredients, and a growing body of safety and efficacy data. However, further research is necessary to evaluate the long-term clinical benefits, optimize manufacturing processes, and ensure compliance with international safety and quality standards. Advances in *in silico* peptide design and database creation will further accelerate the discovery, characterization, and application of peptides in functional foods and nutraceuticals (138–141). Therefore, the future of gut microbiota-targeted BAPs relies on the convergence of personalized nutrition, systems biology enabled by omics, and the evolution of functional food technology. Together, these trends will unlock the therapeutic potential of peptides in shaping microbiome composition and function, advancing the frontiers of nutritional medicine and human wellness.

In conclusion, BAPs represent an emerging class of functional food components with the potential to modulate the gut microbiota and improve host health through interconnected effects on microbial metabolism, immune regulation, epithelial barrier function, and systemic physiology. However, the field is currently transitioning from mechanistic discovery toward clinical translation, and substantial scientific challenges remain. Future research should move beyond identifying new BAPs to understanding the ecological principles governing peptide–microbiota interactions. Instead of viewing BAPs as isolated therapeutic agents, they should be seen as parts of a complex nutritional ecosystem where dietary composition, microbial metabolism, host genetics, and environmental factors together determine biological responses. This systems-level perspective is essential to realize the full therapeutic potential of microbiota-targeted nutritional interventions.

## Glossary

BAPs: Bioactive peptides
ACC: Acetyl-CoA carboxylase
Akt: Protein kinase B
AMP: Antimicrobial peptide
AMPK: AMP-activated protein kinase
BSH: Bile salt hydrolase
CA: Cholic acid
CDCA: Chenodeoxycholic acid
CRC: Colorectal cancer
DCA: Deoxycholic acid
DPP-4: Dipeptidyl peptidase-4
FAS: Fatty acid synthase
FFAR2: Free fatty acid receptor 2
FFAR3: Free fatty acid receptor 3
FXR: Farnesoid X receptor
G6PD: Glucose-6-phosphate dehydrogenase
GIP: Glucose-dependent insulinotropic polypeptide
GLP-1: Glucagon-like peptide-1
GLUT1: Glucose transporter 1
GLUT4: Glucose transporter type 4
GPR41: G protein-coupled receptor 41
GPR43: G protein-coupled receptor 43
GPR109A: G protein-coupled receptor 109A
hBD: Human β-defensin
HDAC: Histone deacetylase
HSL: Hormone-sensitive lipase
IBD: Inflammatory bowel disease
IBS: Irritable bowel syndrome
IECs: Intestinal epithelial cells
IKK: IκB kinase
IL: Interleukin
IRAK: Interleukin-1 receptor-associated kinase
JNK: c-Jun N-terminal kinase
LCA: Lithocholic acid
LPL: Lipoprotein lipase
LPS: Lipopolysaccharide
MAPK: Mitogen-activated protein kinase
MyD88: Myeloid differentiation primary response 88
NF-κB: Nuclear factor kappa B
NOD: Nucleotide-binding oligomerization domain
Nrf2: Nuclear factor erythroid 2-related factor 2
PAMPs: Pathogen-associated molecular patterns
PEG-PCL: Poly (ethylene glycol)-block-polycaprolactone
PepT1: Peptide transporter 1
PI3K: Phosphoinositide 3-kinase
PPAR*α*: Peroxisome proliferator-activated receptor alpha
PPARγ: Peroxisome proliferator-activated receptor gamma
PRRs: Pattern recognition receptors
PYY: Peptide YY
SCFAs: Short-chain fatty acids
TGR5: Takeda G protein-coupled receptor 5 (GPBAR1)
TJs: Tight junctions
TLRs: Toll-like receptors
TMAO: trimethylamine N-oxide
TNF-α: Tumor necrosis factor-alpha
Tregs: Regulatory T cells
ZO-1: Zonula occludens-1
